# Assessment of the direct quantitation of SARS-CoV-2 by droplet digital PCR

**DOI:** 10.1038/s41598-020-75958-x

**Published:** 2020-10-30

**Authors:** Michela Deiana, Antonio Mori, Chiara Piubelli, Salvatore Scarso, Mosè Favarato, Elena Pomari

**Affiliations:** 1 Department of Infectious-Tropical Diseases and Microbiology, IRCCS Sacro Cuore Don Calabria Hospital, Via Don A. Sempreboni, 5 - 37024 Negrar di Valpolicella, Verona, VR Italy; 2 Department of Diagnostics and Public Health, University of Verona, Verona, Italy; 3 Laboratory Medicine, ULSS3 Venetian, Venice, Italy

**Keywords:** SARS-CoV-2, Viral infection

## Abstract

Droplet digital PCR (ddPCR) is a sensitive and reproducible technology widely used for quantitation of several viruses. The aim of this study was to evaluate the 2019-nCoV CDC ddPCR Triplex Probe Assay (BioRad) performance, comparing the direct quantitation of SARS-CoV-2 on nasopharyngeal swab with the procedure applied to the extracted RNA. Moreover, two widely used swab types were compared (UTM 3 mL and ESwab 1 mL, COPAN). A total of 50 nasopharyngeal swabs (n = 25 UTM 3 mL and n = 25 ESwab 1 mL) from SARS-CoV-2 patients, collected during the pandemic at IRCCS Sacro Cuore Don Calabria Hospital (Veneto Region, North-East Italy), were used for our purpose. After heat inactivation, an aliquot of swab medium was used for the direct quantitation. Then, we compared the direct method with the quantitation performed on the RNA purified from nasopharyngeal swab by automated extraction. We observed that the direct approach achieved generally equal RNA copies compared to the extracted RNA. The results with the direct quantitation were more accurate on ESwab with a sensitivity of 93.33% [95% CI, 68.05 to 99.83] and specificity of 100.00% for both N1 and N2. On the other hand, on UTM we observed a higher rate of discordant results for N1 and N2. The human internal amplification control (RPP30) showed 100% of both sensitivity and specificity independent of swabs and approaches. In conclusion, we described a direct quantitation of SARS-CoV-2 in nasopharyngeal swab. Our approach resulted in an efficient quantitation, without automated RNA extraction and purification. However, special care needs to be taken on the potential bias due to the conservation of samples and to the heating treatment, as we used thawed and heat inactivated material. Further studies on a larger cohort of samples are warranted to evaluate the clinical value of this direct approach.

## Introduction

The recent outbreak of severe acute respiratory syndrome coronavirus 2 (SARS-CoV-2) gives rise to a global public health threat (https://covid19.who.int/). SAR-CoV-2 is an enveloped, non-segmented, positive sense RNA virus that is included in the sub-family *Coronavirinae*, subgenus *Sarbecovirus*^1^. As of today, reverse transcriptase real-time PCR (RT-PCR) technology is used as molecular diagnosis for the SARS-CoV-2 and various protocols have been developed and used in the clinical laboratories worldwide^2,3^. The majority of protocols includes the RNA extraction and purification process before RT-PCR as a necessary step for the measurement of viral RNA load, as it isolates the genomic RNA from the viral capsid and removes PCR-inhibitors from the original material^4^. Unfortunately, different extraction kits can provide different amounts of both RNA and inhibitors, hampering the agreement on viral loads and increasing the variability of the data^4,5^. During the SARS-CoV-2 pandemic, it was observed that the relatively low viral load in the throat of patients and the sensitivity limitation of RT-PCR might produce false negatives in the diagnosis^6^. In this context, the droplet digital PCR (ddPCR) might be more appropriate for quantitation of viral loads, as previously reported^7^. The ddPCR allows precise quantitation of nucleic acid copies without the need of a calibration curve and with higher resistance to the amplification inhibitors, compared to the quantitative real-time PCR^8^; some recent studies reported the usage of ddPCR for the quantitation of SARS-CoV-2^9–16^. However, all the described ddPCR procedures included a RNA extraction/purification step, leading to potential amplification errors, due to variable and suboptimal nucleic acid yields^17,18^. To the best of our knowledge, there is only one study reporting the direct quantitation (meaning without RNA extraction) of SARS-CoV-2 by ddPCR, targeting gene E^19^. Concerning the SARS-CoV-2 target genes, previous evidences found that SARS-CoV nucleocapsid (N) region is the optimal target with the highest detection sensitivity^20,21^. Thus, in order to provide new insights on the direct quantitation of SARS-CoV-2 viral loads from swab-derived material, we evaluated the N region (https://www.fda.gov/media/134922/download). In particular, we evaluated the direct ddPCR on two most commonly used nasopharyngeal swabs, the UTM (Universal Transport Medium) 3 mL and the ESwab (Collection and Transport medium) 1 mL (COPAN). In order to evaluate the potential benefits of our ddPCR approach, we compared the ddPCR performance of the direct quantitation with the ddPCR applied to the extracted RNA in both swab types.

## Results

### Limit of detection (LoD) and assessment of variability

**Figure 1:**
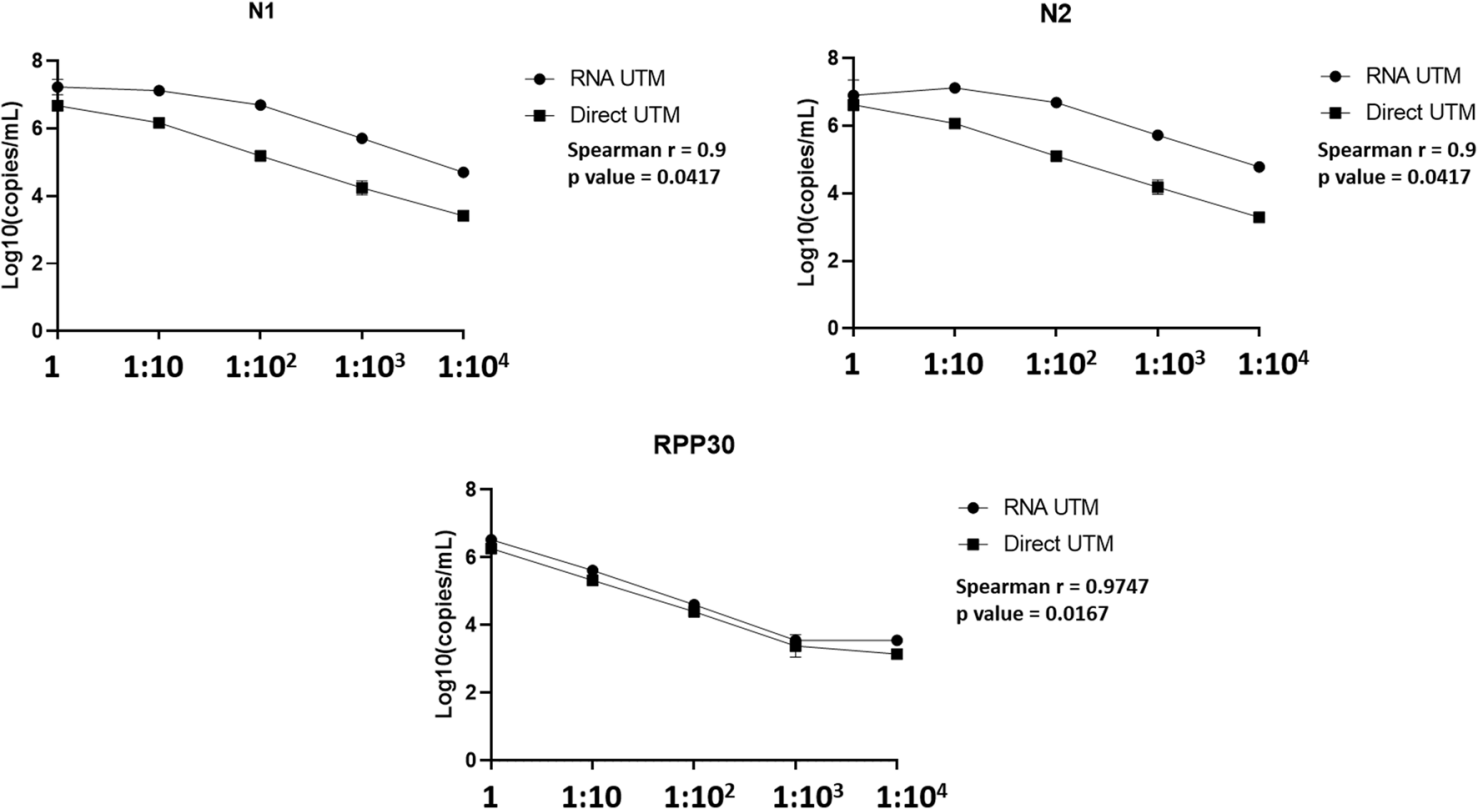
Results of the limit of detection analysis on UTM.

**Figure 2:**
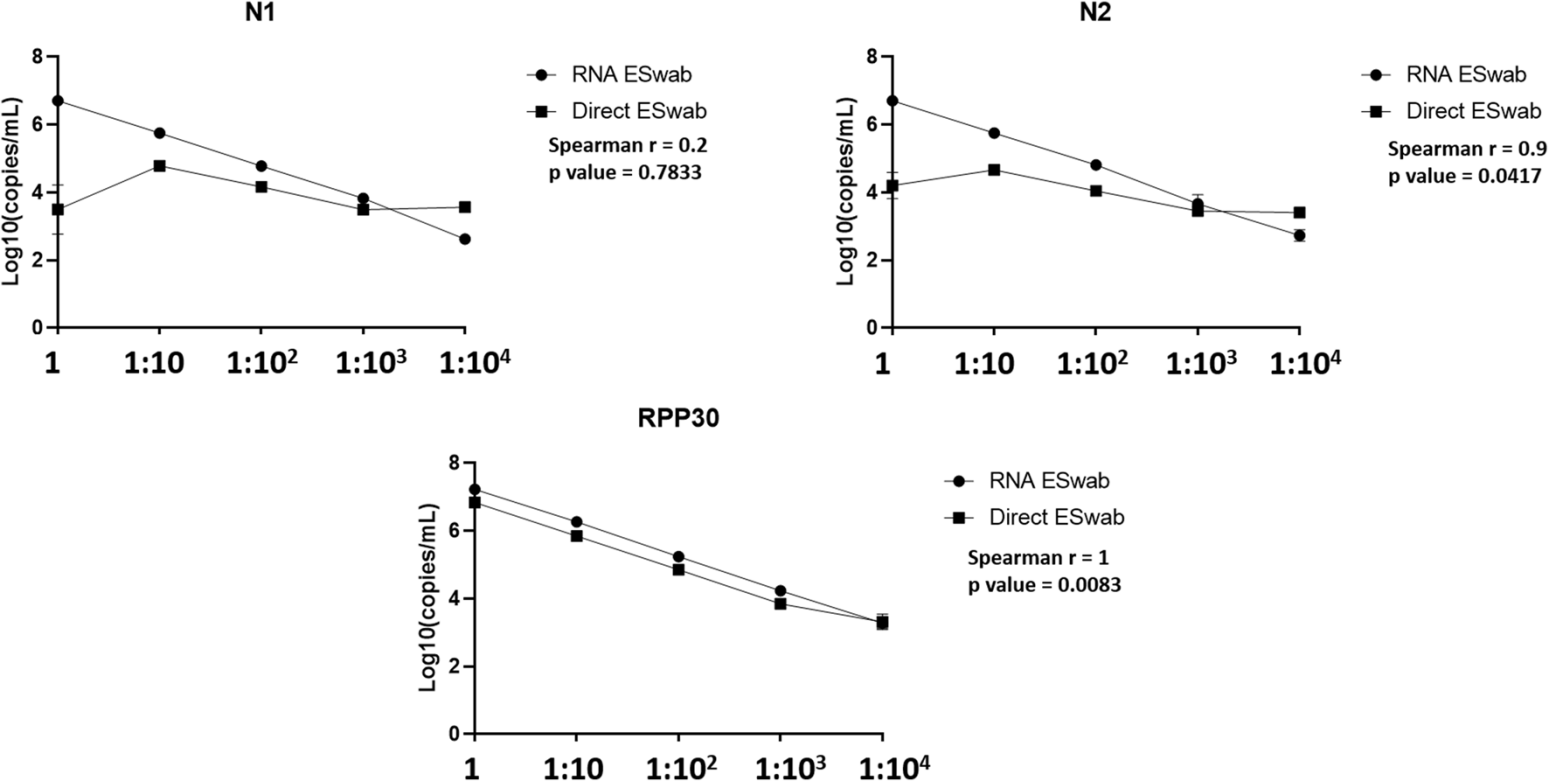
Results of the limit of detection analysis on ESwab.

In order to assess LoD and variability of our direct procedure by ddPCR, we performed a tenfold serial dilution of a patient’s sample for each nasopharyngeal swab type. In particular, we used a confirmed SARS-CoV-2 positive sample by our routine molecular diagnostic testing (for the UTM sample the Ct value was 22.06 for gene N1 and 21.62 for gene N2; for the ESwab sample the Ct value was 20.25 for gene N1 and 21.79 for gene N2). We performed the LoD analysis by ddPCR comparing the direct and the extracted RNA approaches. The viral copies were detected up to dilution 10^3 in all replicates for both swab types by the direct approach (data reported in supplementary material). The measurements of the two approaches are reported in Figs. 1 and 2 with the values obtained by the Spearman correlation. For UTM, the measurement from undiluted material was equal by both approaches, while the undiluted Eswab showed lower viral RNA (N1 and N2) signal than the first dilution. Of note, the internal amplification control (IAC) human RPP30 was generally equal, independently of the swab type. Concerning the variability of data, direct quantitation and quantitation from extracted RNA was equally repeatable, independently of the swab type (data reported in supplementary material). Additionally, the coefficient of variation (CV) using direct quantitation generally did not exceed 10% for both swab types and targets, and was in line with the quantitation of the extracted RNA. On the contrary, the undiluted ESwab showed a higher CV (20%) only for target N1.

### Comparison of ddPCR direct quantitation and quantitation of extracted RNA on patients’ samples

We investigated a number of clinical samples (total n = 50) comparing the extracted RNA and the direct quantitation. For both approaches, the swab material was used undiluted. Table 1 summarizes the results for each swab and target gene. Supplementary material reports detailed data. Thus, in order to evaluate the clinical value of the approaches, we calculated the sensitivity (SE) and specificity (SP) using the results obtained by our routine RT-PCR diagnostic screening as reference values. Based on the data on the single target gene, the SE and SP of direct approach in UTM were respectively 68.75% [95% CI, 41.34 to 88.98] and 90.00% [95% CI, 55.50 to 99.75] for N1 (with the routine RT-PCR Ct value range of 22.02–35.83, Ct mean 32.77). For N2, the SE and SP were 66.67% [95% CI, 38.38 to 88.18] and 90.00% [95% CI, 55.50 to 99.75] respectively (with the routine RT-PCR Ct value range of 21.62–37.5, Ct mean 32.52). Of note, we observed that the discordant results for N1 and N2 had Ct value ≥ 34 (by the routine RT-PCR).

**Table 1:** ddPCR results on the cohort.

Swab	Target	Positives				Negatives			
		N positives/N tested by RNA extraction	N positives/N tested by direct quantitation	RNA vs direct Log (copies/mL) Mean ± SD	RNA vs direct *P* value	N negatives/N tested by RNA extraction	N negatives/N tested by direct quantitation	RNA vs Direct Log(copies/mL) Mean ± SD	RNA vs Direct *P* value
UTM	N1	14/15	11/15	5.36 (± 1.56) vs 3.72 (± 1.22)	0.0059	10/10	9/10	nd vs 3.92	> 0.9999
	N2	14/15	10/15	4.50 (± 1.22) vs 4.00 (± 1.09)	0.0742	10/10	9/10	nd vs 3.47	> 0.9999
	*RPP30*	15/15	15/15	6.12 (± 0.54) vs 5.96 (± 0.64)	0.0084	0/10	0/10	5.18 (± 0.27) vs 4.77 (± 0.29)	0.0020
ESwab	N1	14/15	14/15	4.20 (± 1.27) vs 3.76 (± 0.64)	0.3258	10/10	10/10	nd vs nd	> 0.9999
	N2	10/15	14/15	4.75 (± 1.07) vs 3.88 (± 0.49)	0.0840	10/10	10/10	nd vs nd	> 0.9999
	*RPP30*	15/15	15/15	6.44 (± 0.68) vs 6.18 (± 0.75)	0.2769	0/10	0/10	4.94 (± 0.47) vs 5.22 (± 0.62)	0.0195

If we considered a combination of N1 and N2 results, the SE increased to 80% [95% CI, 51.91 to 95.67] and the SP to 93.33% [95% CI, 68.05 to 99.83]. In ESwab direct approach, considering N1 and N2 independently, the SE and SP were respectively 93.33% [95% CI, 68.05 to 99.83] and 100.00% for both N1 (with RT-PCR Ct range of 16.81–38.77, Ct mean 27.36) and N2 (with RT-PCR Ct range of 17.73–37.58, Ct mean 28.42). If we combined N1 and N2, the SE and SP were 100%. On the other hand, using the extracted RNA from the same patient’s samples, the sensitivity (SE) and specificity (SP) in UTM were respectively 93.33% [95% CI, 68.05 to 99.83] and 100% for both N1 and N2. In ESwab the SE was 93.33% [95% CI, 68.05 to 99.83] for N1 and 66.67% [95% CI, 38.38 to 88.18] for N2, while and the SP was 100% for both gene targets. When we combined N1 and N2, the SE was 93.33% [95% CI, 68.05 to 99.83] and SP 100% for both UTM and ESwab. The SE and SP were 100% for RPP30 independently of swab types and approaches. In summary, despite some discordant results, the measurements of RNA copies were generally equal for both viral and human RNA between the direct and the RNA extracted.

### Comparison of ddPCR results between UTM and ESwab

We compared the results obtained using ddPCR on UTM swabs with those obtained on ESwab in our cohort (Table 2). No statistically significant differences were detected between the UTM and ESwab in terms of measured copy numbers, for both the direct and extracted RNA approaches, with the exception of N1 quantitation in extracted RNA, in which the UTM method showed a slightly higher signal (*p* = 0.0105, Wilcoxon test). However, the data need to be confirmed with a larger number of measurements.

**Table 2:** Comparison of ddPCR results on the SARS-CoV-2 positive (N = 15 UTM and N = 15 ESwab) and negative (N = 10 UTM and N = 10 ESwab) subjects of the cohort.

ddPCR	Target	Positives		Negatives	
		UTM vs ESwab Log(copies/mL) Mean ± SD	UTM vs ESwab *P* value	UTM vs ESwab Log(copies/mL) Mean ± SD	UTM vs ESwab *P* value
RNA	N1	5.36 (± 1.56) vs 4.20 (± 1.27)	0.0105	nd vs nd	na
	N2	4.50 (± 1.22) vs 4.75 (± 1.07)	0.7148	nd vs nd	na
	RPP30	6.12 (± 0.54) vs 6.44 (± 0.68)	0.1876	5.18 (± 0.27) vs 4.94 (± 0.47)	0.3750
Direct	N1	3.72 (± 1.22) vs 3.76 (± 0.64)	0.6953	nd vs nd	> 0.9999
	N2	4.00 (± 1.09) vs 3.88 (± 0.49)	0.6523	3.47 (na) vs nd	> 0.9999
	RPP30	5.96 (± 0.64) vs 6.18 (± 0.75)	0.4545	4.77 (± 0.29) vs 5.22 (± 0.62)	0.1055

Na: not applicable; nd: not detected. Wilcoxon Test *p* values are reported.

## Discussion

As of today, few studies reported the use of ddPCR for a more sensitive SARS-CoV-2 detection compared to RT-PCR^12,14–16^. To our knowledge, this is the first report of direct quantitation of SARS-CoV-2 RNA performed on a consistent number of clinical samples and comparing two different nasopharyngeal swabs. Indeed, the RNA extraction from nasopharyngeal swabs of patients affected by SARS-CoV-2 might slacken the diagnostic process due principally to the shortage of reagents for the RNA extraction. To overcome this issue, direct protocols from swab samples before conducting molecular diagnostics have been assessed and reported^19,22,23^. In our study, we analysed results obtained by ddPCR using an assay based on the Center for Disease Control and Prevention (CDC) recommended targets, directly on material derived from two different nasopharyngeal swab types (UTM and ESwab). The results were compared with those obtained from the RNA extracted from the same swabs. In particular, the data obtained from the human IAC (RPP30), showed that the direct quantitation approach achieved generally equal RNA copies compared to those from the extracted RNA, independently of the swab. On the other hand, for the viral load (N1 and N2 genes), the ddPCR measurements showed that the direct quantitation was generally equal to that obtained from the RNA extracts, but when we performed the limit of detection on ESwab, we observed that the undiluted material might be underestimated. One possible explanation for the different results between UTM and ESwab, could be due to the fact that the varying volume of swab media might denote different amount of both inhibitors and viral capsid proteins influencing the direct quantitation^17^. In order to overcome this issue, we could hypothesize that the introduction to our procedure of a supplementary pre-treatment using proteinase K^19^ could be helpful. Moreover, we used two different viral targets (N1 and N2), but future study targeting additional viral regions could be valuable in order to minimize this potential bias and to increase the chance of amplification^17^. In our work, other potential bias of ddPCR quantities should be taken into consideration as we used thawed material and we chose to pre-heat samples for viral inactivation instead of using chemical treatment. Indeed we avoided to use the most commonly used guanidinium to circumvent possible cause of inhibition of the amplification^24,25^. However, the heating step was used only for the direct quantitation and it could be another possible cause of underestimation of the amplification as reported recently^26^.

To conclude, with this work we have demonstrated that our procedure allows the direct quantitation of SARS-CoV-2 RNA. Our ddPCR procedure is simple and direct, avoiding the possible limitations due to the lack of commercial kits for the extraction. The strategy that we proposed does not require great changes of the workflow for laboratories performing the CDC assay. Concerning the performance of direct quantitation on different swab-derived material, although the data obtained on UTM showed generally equal measurement to the RNA extracted, a low SE was found on our cohort. The results from ESwab were more accurate in terms of SE and SP on the cohort samples, but with potential higher amount of inhibitors. Thus, a larger number of specimens and data from other laboratories are needed to evaluate the clinical value of the direct procedure. Further investigations will be necessary focusing on the assessment of the performance of the direct RNA quantitation *i)* on fresh swab-derived material and *ii)* using additional viral targets.

## Methods

### Setting of the study

**Figure 3:**
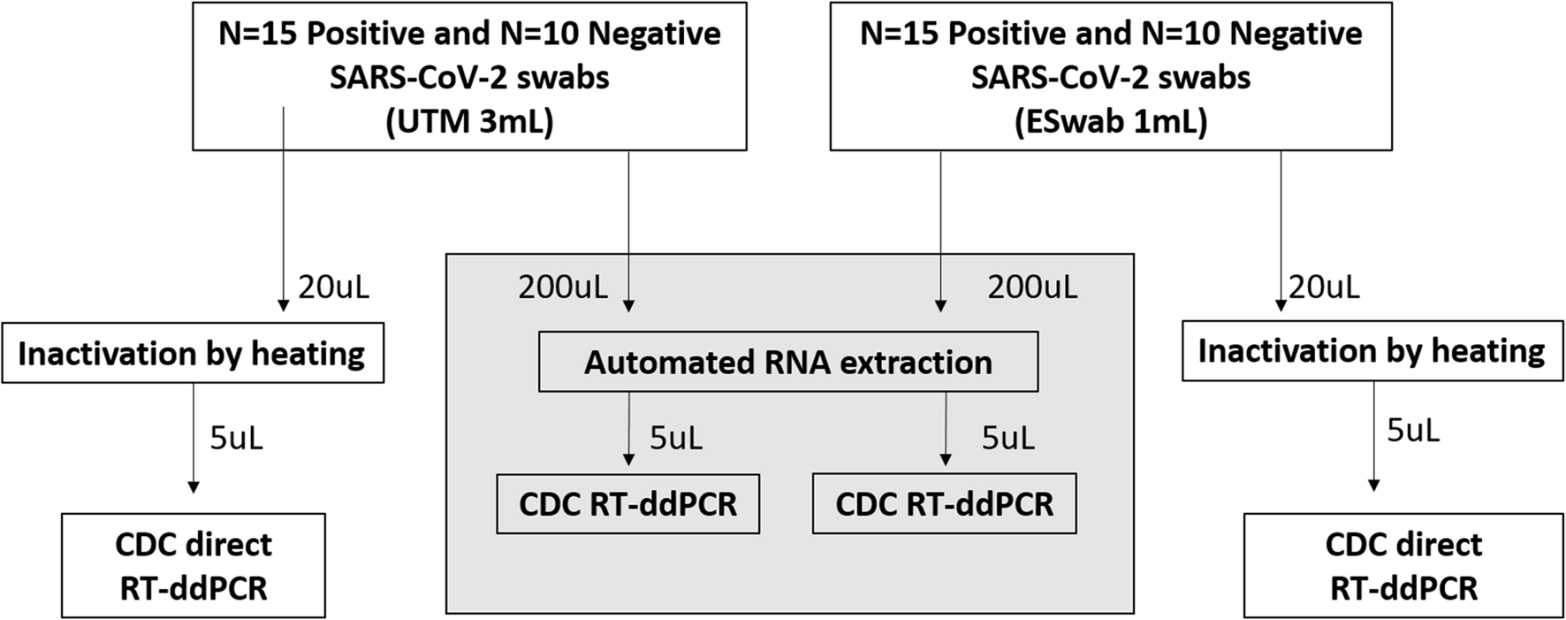
Flow chart of the study.

A total of 50 anonymized samples were used. All the samples were previously screened by RT-PCR with our routine diagnostic testing based on the CDC protocol (N1 and N2 genes) (https://www.fda.gov/media/134922/download). N = 30 samples were positive and N = 20 were negative to SARS-CoV-2. We analysed samples collected using two different nasopharyngeal swab types: N = 15 positive and N = 10 negative in UTM 3 mL (COPAN) and N = 15 positive and N = 10 negative in ESwab 1 mL (COPAN) (Fig. 3). For the ddPCR analysis performed in the preset study, we used aliquots of samples stored at -80 °C. The aliquots were thawed and used for both the automated extraction of RNA and the direct quantitation. All the procedures were performed in BLS2 laboratories, according to the biosafety guidelines for handling and processing specimens associated with Coronavirus Disease 2019 (COVID-19) (https://www.cdc.gov/coronavirus/2019-nCoV/lab/lab-biosafety-guidelines.html#guidance).

### Automated RNA extraction

RNA was isolated from 200 μl of nasopharyngeal swab medium by the Nextractor NX-48 robot, using the NX-48S Viral NA Kit (Genolution Inc.), according to the manufacturer’s instructions. Samples were eluted in 50μL of elution buffer. The isolated RNA was immediately used by ddPCR.

### Heat inactivation for direct quantitation

For the direct ddPCR, 20 μl of thawed medium from UTM and ESwab were added in a 96-well plate and incubated at 56 °C for 10 min^27–29^ (https://www.who.int/csr/sars/survival_2003_05_04/en/), followed by 4 °C for 5 min and then immediately used by ddPCR.

### One step reverse transcriptase—ddPCR

The ddPCR procedure was performed following the manufacturer’s instructions of the 2019-nCoV CDC ddPCR triplex probe assay (dEXS28563542, Bio-Rad). The PCR reaction mixture was assembled as follows: One-Step supermix 2 × for probe (no dUTP) (Bio-Rad), 20 × Assay (for N1, N2, RPP30 detection), reverse transcriptase 20U/μl, RNase free water 7 μl, and RNA template 5 μl or inactivated swab medium 5 μl, in a final volume of 22 μl. Then, QX200 droplet generator (Bio-Rad) was used to convert 20 μl of each reaction mix into droplets. The Droplet-partitioned samples were transferred to a 96-well plate, sealed and processed in a C1000 touch Thermal Cycler (Bio-Rad) under the following cycling protocol: 25 °C for 3 min, 50 °C for 60 min for reverse transcription, 95 °C for 10 min for enzyme activation, 95 °C for 30 s for denaturation and 55 °C for 60 s for annealing/extension for 40 cycles, 98 °C 10 min for enzyme deactivation followed by infinite 4-degree hold. The amplified samples were then transferred and read in the FAM and HEX channels using the QX200 reader (Bio-Rad). The experiments were performed using a negative control (no template control, NTC) and a positive control (a patient’s sample confirmed positive by RT-PCR with our routine diagnostic testing). The reactions with less than 10,000 droplets and discordant results were repeated. Data were analysed using the QuantaSoft™ v1 AnalysisPro Software (Bio-Rad) and expressed as Log_10_ (copies/mL).

### Limit of detection analysis

For each nasopharyngeal swab, we used a patient’s sample (confirmed positive by RT-PCR with our routine diagnostic testing) to generate tenfold serial dilutions for a total of five points of both RNA extracted and swab-derived material. Each dilution point was analysed by ddPCR in triplicate and the repeatability intra-assay was assessed. Results were expressed as Log_10_ (copies/mL).

### Statistics

The statistical analyses and graphical representations were performed by GraphPad Prism 8. Data are reported as mean ± SD. Spearman’s correlation was performed between measurements, due to the small sample size. Paired non parametric Wilcoxon Test was performed to compare the two approaches, ddPCR on RNA extracted and direct ddPCR on the swab-derived material UTM and ESwab. A *p* value ≤ 0.05 was considered statistically significant.

### Ethical statement

The study (No. 39528/2020 Prog. 2832CESC) was approved by the competent Ethics Committee for Clinical Research of Verona and Rovigo Provinces. Written informed consent was obtained from the patients and all research was performed in accordance with relevant guidelines/regulations.

## Supplementary information

Supplementary information

## Data Availability

All data generated or analysed during this study are included in this published article (and its supplementary information files).
